# Effects of steroidal and nonsteroidal aromatase inhibitors on markers of bone turnover in healthy postmenopausal women

**DOI:** 10.1186/bcr1757

**Published:** 2007-08-10

**Authors:** Paul E Goss, Peyman Hadji, Milayna Subar, Paula Abreu, Torben Thomsen, Jose Banke-Bochita

**Affiliations:** 1 Massachusetts General Hospital, 55 Fruit Street, LRH 302, Boston, MA, 02114 USA; 2 Philipps-University of Marburg, Philipps-Universität, Biegenstr. 10, 35032, Marburg, Germany; 3 Pfizer Inc., 235 East 42nd Street, New York New York 10017-5755 USA; 4 CRS Clinical Research Services, Moenchengladbach GmbH, Hindenburgstrasse 306 41061 Moenchengladbach, Germany; 5 PAREXEL GmbH, Klinikum Westend, Haus 18, Spandauer Damm 115, D-14050 Berlin, Germany

## Abstract

**Introduction:**

In contrast to nonsteroidal aromatase inhibitors, the steroidal aromatase inactivator exemestane does not have detrimental effects on bone in animal models. This study was designed to compare the effects of exemestane with the nonsteroidal aromatase inhibitors anastrozole and letrozole on serum and urine levels of biomarkers of bone turnover in healthy postmenopausal women.

**Methods:**

Changes in the concentrations of bone-turnover markers, estrogens, and lipids were assessed after daily administration of exemestane (25 mg), letrozole (2.5 mg), anastrozole (1 mg), or placebo for 24 weeks in healthy postmenopausal women. The primary end point was the percentage change from baseline in bone-turnover-marker levels at week 24. The baseline-adjusted area under the curve (AUC) for weeks 0–12 and 0–24 was calculated to evaluate changes in bone turnover over time, rather than at discrete time points.

**Results:**

Seventy-four (88%) of 84 randomized subjects were evaluable for bone-marker assays. Reductions in plasma estrogen levels and increases in bone-resorption markers were comparable for each aromatase inhibitor. Uniquely, exemestane consistently increased the percentage change from baseline in the level of serum procollagen type I N-terminal propeptide (PINP), a marker of bone formation, at week 24. In the active-treatment groups, the baseline-adjusted AUC at weeks 0–12 and 0–24 for PINP was significantly greater for exemestane than the other aromatase inhibitors.

**Conclusion:**

Exemestane increased serum levels of the bone-formation marker PINP after 24 weeks, suggesting a specific bone-formation effect related to its androgenic structure. Potential effects on cortical bone and reduced fracture risk must be verified in a comparative clinical trial.

## Introduction

Aromatase inhibitors (AIs), which potently suppress estrogen synthesis in postmenopausal women, are increasingly used in the treatment of early-stage breast cancer to prevent disease recurrence. AIs reduce the plasma estrogen concentration by suppressing peripheral estrogen synthesis and might also suppress estrogen synthesized locally by aromatase in bone [1]. Because osteoporosis and the concomitant fracture risk are potentially serious adverse effects of AIs, a better understanding of their effects on bone is necessary.

Two classes of AIs, nonsteroidal and steroidal, are currently used in the treatment of breast cancer patients. After 1 year of treatment, the nonsteroidal AI anastrozole was reported to increase markers of bone turnover by 12.2–20.8% in postmenopausal women who had early breast cancer [2]. In the same study, after 1 and 2 years, anastrozole induced decreases of 2.6% and 4.0%, respectively, in lumbar spine bone mineral density (BMD) and 1.7% and 3.2%, respectively, in total hip BMD [3]. The nonsteroidal AI letrozole has similar effects. In a study in healthy late-postmenopausal women, letrozole increased markers of bone turnover by 15% after 6 months [4], and in a similar study, there was a 25% increase in a marker of bone resorption after 3 months of letrozole treatment [5]. Preliminary results from a large, multicenter, open-label trial demonstrated that after 1 year of treatment with letrozole, lumbar spine and total hip BMDs decreased by 2.6% and 2.1%, respectively [6].

In a preclinical model, the steroidal aromatase inactivator exemestane showed no detrimental effects on markers of bone turnover or BMD. Moreover, exemestane was protective against bone loss that occurred following ovariectomy in rats, whereas letrozole was not [7]. In the clinical setting, postmenopausal women with early breast cancer treated using exemestane (25 mg daily) or placebo for 2 years experienced a mean annual bone loss of 2.2% vs 1.8% (*P *= 0.57), respectively, in the lumbar spine and 2.7% vs 1.5% (*P *= 0.024), respectively, in the femoral neck. Exemestane also significantly increased levels of biomarkers for bone resorption and formation [8]. Because of the lack of any significant difference in the change in BMD in the lumbar spine, an area with frequent compression fractures, the clinical implication of a 1.2% (90% confidence interval (CI), 0.3–2.1%) difference in femoral neck BMD between the exemestane-treated group and the placebo group has been questioned [9]. Although these data support the possibility that exemestane might be associated with an attenuation of bone loss, they do not fully resolve questions regarding the role or mechanism of exemestane in bone turnover.

Other clinical studies have also reported unfavorable bone effects of exemestane, anastrozole, and letrozole [10-15], including an increased risk of fractures using anastrozole [16] and exemestane [14] compared with tamoxifen. However, the prior administration of tamoxifen – which is bone protective – in several of these studies makes interpretation of these findings difficult.

To date, there are no comparative data on the effects of the different AIs on bone. This study was conducted to compare the effects of the steroidal AI exemestane with the nonsteroidal AIs anastrozole and letrozole or placebo on serum and urine levels of biomarkers of bone turnover in healthy postmenopausal women.

## Materials and methods

This randomized, single-blind, placebo-controlled exploratory study investigated the effect of low plasma estrogen levels, induced by AIs, on markers of bone turnover in 80 healthy postmenopausal women during 24 weeks of outpatient treatment using exemestane, letrozole, anastrozole, or placebo. All patients provided written informed consent. The study was conducted at two sites in Germany in accordance with the ethical principles that originated in the Declaration of Helsinki.

### Patient selection and treatment

Healthy postmenopausal volunteers between 50 and 75 years of age were enrolled. Eligible subjects were at least 1 year postmenopause (defined as no menstrual bleeding for at least 1 year before screening and documented luteinizing and follicle-stimulating hormone levels within the postmenopausal range), had a body-mass index of 19–35 kg/m^2^, and had a body weight of 55–95 kg. All subjects had a normal BMD for their age, as confirmed by quantitative ultrasonometry (QUS).

Exclusion criteria included current or previous use of bisphosphonates or any other drug known to affect bone metabolism (including hormone-replacement therapy and statins) within 1 month before study entry, use of any medication within 2 weeks or five half-lives of the medication (whichever was longer) before study entry, subclinical hyperthyroidism or other metabolic disorder, any form of osteoporosis (as measured by BMD or QUS testing), and a history of bilateral oophorectomy.

Eligible subjects were randomized 1:1:1:1 to receive 24 weeks of treatment with anastrozole (1 mg), exemestane (25 mg), letrozole (2.5 mg), or placebo. Placebo tablets were identical in appearance to exemestane. Each treatment was administered orally, once daily after breakfast. Follow-up was continued until 12 weeks after the last dose of study medication (36 weeks).

### Study end points

Subjects were evaluated at baseline and 2, 4, 8, 12, 16, 20, 24, and 36 (follow-up) weeks. At each visit, clinical and laboratory safety tests were performed and blood and urine samples were obtained for assessment of the study end points.

The primary study end point was the percentage change from baseline to week 24 in bone-turnover biomarkers, including bone-formation markers (bone alkaline phosphatase (BAP) and procollagen type I N-terminal propeptide (PINP)) and bone-resorption markers (serum C-terminal telopeptide of type I collagen (S-CTx), urine-adjusted urinary (U-)CTx, and urine-adjusted urinary N-terminal telopeptide of type I collagen (U-NTx)).

Predefined secondary end points included the percentage change from baseline in concentrations of bone-turnover biomarkers at weeks 12 and 36 (12 weeks after completion of treatment), change in the baseline-adjusted area under the curve (AUC) for weeks 0–12 and 0–24 for bone-turnover markers, percentage of baseline estrogen concentrations (estradiol (E_2_), estrone (E_1_), and estrone sulfate (E_1_S)) at weeks 12, 24, and 36, percentage change from baseline in lipid parameters (total cholesterol, low-density lipoprotein cholesterol, high-density lipoprotein cholesterol, and triglycerides) at weeks 12, 24, and 36, and safety.

Adverse events were monitored by means of verbal probes and spontaneous reports and reported using the National Cancer Institute Common Toxicity Criteria (NCI CTC) medical terminology and grading system, version 2.0. Patients experiencing adverse events that were considered to be causally related to study medication were followed up until resolution of the event.

### Sample collection and handling

Blood and urine samples were obtained at baseline and 2, 4, 8, 12, 16, 20, 24, and 36 (follow-up) weeks. Blood samples for estrogen measurement were collected into prechilled heparinized tubes. Baseline levels were defined as the mean of observations taken on study days -2, -1, and 0. Subjects were instructed to consume nothing by mouth after midnight on the day before scheduled visits.

Serum was harvested from blood samples, which were drawn in the morning before drug dosing and after an overnight fast. Second-void urine samples were collected in the morning before dosing and after an overnight fast. Serum, plasma, and urine samples were stored at -20°C until processed.

### Laboratory methods

All assays were performed in centralized laboratories under blinded conditions. Assays for CTx and NTx were performed on urine samples. Urinary assay values were corrected for urinary dilution by urinary creatinine analysis [17]. U-CTx and U-NTx levels were adjusted for renal function according to the level of urinary creatinine. Concentrations of BAP, CTx, PINP, and lipids were measured in serum samples. Estrogen levels were measured in plasma. The levels of bone-turnover markers, lipids, and estrogens were evaluated by radioimmunoassay, ELISA, or automated tests using commercially available kits as specified by the manufacturers (Table 1). For the estrogen assays, the radioimmunoassays were performed on plasma samples previously purified by HPLC to remove any potential interference by exemestane and its metabolites [18].

**Table 1:** Laboratory methods for assay of bone-turnover markers, lipids, and estrogens

**Parameter**	**Method**	**Manufacturer**
**Bone resorption**		
Adjusted U-CTx	Enzyme immunoassay test using the CrossLaps^® ^ELISA kit	Nordic Bioscience Diagnostics A/S, Herlev, Denmark
S-CTx	Electrochemoluminescence immunoassay technique using Elecsys 2010 automate analyzer	Roche Diagnostics, Basel Switzerland
Adjusted U-NTx	Competitive immunoassay technique using the NTx reagent pack kit	Ortho-Clinical Diagnostics, Amersham, UK
**Bone formation**		
BAP	Ostase Assay using Access Immunoassay System	Beckman Coulter, Villepinte, France
PINP	Electrochemoluminescence immunoassay technique using Elecsys 2010 automate analyzer	Roche Diagnostics
**Lipids **(total cholesterol, LDL cholesterol, HDL cholesterol, and triglycerides)	Validated HPLC–RIA technique using Bayer Advia Reagent Packs. Assayed on an ADVIA^® ^1650 chemistry system analyzer	Bayer Diagnostics, Bayer Inc., Tarrytown, NY, USA
**Estrogen **(E_1_, E_2_, and E_1_S)	Validated HPLC–RIA technique	Aster-Cephac, Paris, France

BAP, bone alkaline phosphatase; E_1_, estrone; E_1_S, estrone sulfate; E_2_, estradiol; ELISA, enzyme-linked immunosorbent assay; HDL, high-density lipoprotein; HPLC–RIA, high-performance liquid chromatography radioimmunoassay; LDL, low-density lipoprotein; PINP, procollagen type I N-terminal propeptide; S-CTx, serum C-terminal telopeptide of type I collagen; U-CTx, urine-adjusted urinary C-terminal telopeptide of type I collagen; U-NTx, urine-adjusted urinary N-terminal telopeptide of type I collagen.

### Statistical analysis

This was an exploratory study, and no reliable comparable bone-turnover-marker data are available in this study population. The sample size was calculated according to an effect size, defined as the expected difference in group means to the within-group standard deviation. A sample size of 20 within each of the four treatment groups was calculated to have an 80% power to detect an approximate effect size of 0.9 at the 0.05 level. The current analysis was performed in the evaluable population, which included all randomized subjects who received at least one dose of study medication, had evaluable data from the baseline and week 12 assessments at least, and maintained suppression of estrogen throughout the study.

Descriptive statistics, including mean, standard deviation, median, and range for continuous variables and frequency and percentage for categorical variables, were used for all demographics, baseline characteristics, and pretreatment conditions, as appropriate. Percentage change from baseline in bone-turnover markers and lipid profiles and percentage of baseline estrogen concentrations were also summarized by assessment time and treatment arm using descriptive statistics. A one-way analysis of variance model was used to test baseline and demographic characteristics for homogeneity of the populations in each of the four treatment groups. The Kruskall–Wallis test was used to exclude differences in the baseline values of bone-turnover markers, plasma estrogen concentrations, and lipid profiles between the three active treatment groups and compare the percentage change in these parameters from baseline. Values reported as below the limit of quantification for markers of bone turnover (<2 for creatinine and ≤28 for U-NTx) were treated as missing data.

The baseline-adjusted AUC (henceforth referred to as AUC for weeks 0–12 and 0–24 was calculated for all bone-turnover markers to evaluate accumulated changes in bone turnover over time, rather than at discrete time points [19]. The AUC was calculated as follows, using BAP as an example: the baseline-adjusted AUC_0-t week _was calculated as the AUC_BAP-t* _baseline concentration of BAP, where *t *= 12 or 24 weeks. The AUC_BAP(0-t) _was then calculated by summing the partial AUCs using the trapezoidal rule. The AUC was summarized by assessment time and treatment arm by means of descriptive statistics.

## Results

**Figure 1:**
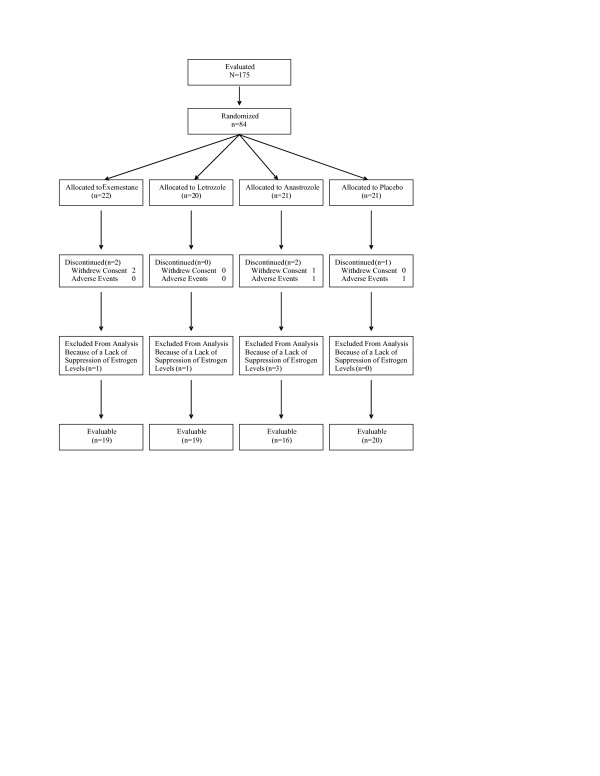
Patient disposition.

One hundred and seventy-five subjects were screened. A total of 84 subjects were randomized to one of four treatment groups and included in the safety evaluation. All randomized subjects received at least one dose of study medication. Estrogen and lipid levels were reported in the modified intent-to-treat (mITT) population (*n *= 79), which excluded five subjects who discontinued treatment early owing to withdrawal of consent (one anastrozole- and two exemestane-treated subjects) and adverse events (one placebo- and one anastrozole-treated subject). Estrogen levels were not suppressed in five subjects (one exemestane-, one letrozole-, and three anastrozole-treated subjects). Estrogen levels returned to baseline values after initial suppression to below the limit of quantification in two additional subjects who were presumed to be noncompliant. Because the intent of the study was to assess differences among AIs in the presence of low plasma levels of estrogen, these subjects were excluded from the bone-marker analyses; therefore, the evaluable population for bone-marker data from baseline comprised 74 subjects. Bone-marker data for the two additional subjects were excluded from the analysis from the time the estrogen levels increased (weeks 16 and 20, respectively). Patient disposition is summarized in Figure 1.

Baseline characteristics were comparable among the four treatment groups in the safety and mITT populations. Baseline demographics for the evaluable population are summarized in Table 2. All subjects were white women who had experienced a natural menopause; no subjects had undergone ovariectomy. The overall mean (standard deviation) time since the onset of menopause was 11.7 (6.7) years (range, 1–28 years). Baseline concentrations of bone-turnover markers, estrogens, and lipids were similar among the treatment groups (Table 2).

**Table 2:** Baseline characteristics and baseline bone turnover values in the safety population

	**Mean (SD)** **Median (min., max.)**			
	
**Parameter**	**Exemestane (*n *= 22)**	**Letrozole (*n *= 20)**	**Anastrozole (*n *= 21)**	**Placebo (*n *= 21)**
**Baseline characteristics**				
Age, years	61 (4.7) 61 (52, 72)	60 (4.7) 60 (47, 69)	57 (5.1) 56 (50, 64)	59 (4.2) 60 (52, 65)
Weight, kg	69 (7.5) 70 (56, 85)	72 (9.4) 70 (59, 95)	66 (7.4) 68 (55, 83)	72 (13) 73 (45, 102)
Height, cm	165 (6.4) 165 (156, 178)	162 (4.7) 163 (149, 170)	163 (5.0) 163 (154, 172)	164 (4.5) 164 (155, 173)
BMI, kg/m^2^	26 (2.8) 25 (21, 32)	27 (3.5) 28 (22, 35)	25 (2.9) 24 (20, 32)	27 (4.8) 27 (18, 38)
Time since menopause, years	13 (8.1) 12 (1.0, 28)	12 (7.4) 9.0 (3.0, 25)	12 (6.6) 8.5 (4.0, 25)	10 (5.3) 10 (3.0, 21)
Baseline bone-turnover values*				
BAP, ng/ml	16 (7.8) 13 (7.4, 38)	12 (3.3) 11 (6.1, 19)	16 (5.4) 16 (6.8, 25)	14 (3.7) 14 (5.4, 19)
S-CTx, ng/ml	0.51 (0.21) 0.49 (0.23, 0.94)	0.39 (0.12) 0.41 (0.18, 0.59)	0.54 (0.16) 0.58 (0.21, 0.79)	0.41 (0.14) 0.39 (0.16, 0.66)
PINP, ng/ml	57 (25) 55 (21, 106)	44 (12) 41 (25, 74)	53 (20) 51 (13, 92)	48 (19) 45 (23, 96)
U-CTx, μg/mmol Cr	289 (143) 237 (140, 725)	234 (66) 242 (112, 358)	338 (98) 322 (133, 550)	256 (68) 241 (174, 383)
U-NTx, nmol BCE/mmol Cr	61 (31) 55 (19, 140)	53 (16) 55 (27, 85)	66 (24) 63 (40, 128)	61 (22) 54 (27, 103)
**Baseline estrogen concentrations (pg/ml)**				
E_1_	34 (15) 31 (11, 72)	30 (11) 28 (13, 51)	31 (12) 28 (14, 61)	30 (11) 27 (10, 61)
E_2_	4.7 (2.5) 4.4 (1.1, 13)	4.8 (2.1) 4.2 (1.8, 8.6)	6.3 (7.0) 3.9 (1.9, 32)	4.5 (2.1) 3.9 (1.9, 9)
E_1_S Estrone sulfate	221 (150) 189 (58, 757)	269 (222) 193 (79, 1075)	252 (199) 197 (64, 896)	274 (249) 211 (62, 1156)
**Baseline lipid concentrations (mmol/L)**				
Total cholesterol	5.9 (0.70) 6 (5, 7)	6.1 (0.65) 6 (5, 8)	6.2 (0.82) 6 (4, 8)	5.8 (0.73) 6 (4, 8)
LDL cholesterol	4 (0.8) 4 (2, 6)	4 (0.9) 4 (3, 7)	4 (0.8) 4 (3, 6)	4 (0.7) 4 (2, 6)
HDL cholesterol	1.5 (0.41) 1.4 (0.90, 2.6)	1.5 (0.43) 1.5 (0.87, 2.2)	1.4 (0.39) 1.4 (0.83, 2.4)	1.5 (0.31) 1.4 (1.1, 2.3)
Triglycerides	1.4 (0.81) 1.1 (0.67, 3.7)	1.3 (0.44) 1.2 (0.72, 2.2)	1.3 (0.48) 1.2 (0.54, 2.5)	1.1 (0.42) 1.0 (0.52, 2.1)

*The baseline value was calculated as the mean of three baseline measurements.

BAP, bone alkaline phosphatase; BCE, bone collagen equivalent; BMI, body-mass index; Cr, creatinine; E_1_, estrone; E_1_S, estrone sulfate; E_2_, estradiol; HDL, high-density lipoprotein; LDL, low-density lipoprotein; max; min; PINP, procollagen type I N-terminal peptide; S-CTx, serum C-terminal telopeptide of type I collagen; SD, standard deviation; U-CTx, urine C-terminal telopeptide of type I collagen; U-NTx, urine N-terminal telopeptide of type I collagen.

### Bone turnover

Baseline concentrations of bone-turnover markers in the evaluable population were similar among the treatment groups (Table 2). The effects of each treatment on bone-turnover markers are summarized in Table 3.

**Table 3:** Summary of primary and secondary efficacy results for bone-turnover markers in the evaluable population

**Bone-turnover marker**	**Exemestane (*n *= 19)**	**Letrozole (*n *= 19)**	**Anastrozole (*n *= 16)**	**Placebo (*n *= 20)**
Primary efficacy measure: percentage change from baseline to week 24*				
BAP	3.0 (-11, 24)	-0.58 (-9.4, 7.1)	-4.5 (-7.0, 4.5)	-0.88 (-11.8, 8.1)
S-CTx	21 (6.9, 34)	31 (12, 69)	9.3 (-7.7, 34)	12 (-8.7, 26)
PINP	24 (11, 30)	5.7 (-1.1, 12)	6.3 (-9.3, 19)	6.1 (-11, 16)
U-CTx	22 (10, 35)	34 (19, 74)	1.7 (-5.6, 29)	15 (1.5, 29)
U-NTx	-8.5 (-30, 1.9)	10 (-1.5, 20)	-1.5 (-26, 24)	1.7 (-13, 19)
Secondary efficacy measure: percentage change from baseline to week 12*				
BAP	-1 (-5.7, 8.0)	-4.3 (-7.5, 7.3)	-7.3 (-17, 3.7)	-0.99 (-6.0, 3.3)
S-CTx	18 (10, 37)	18 (7.2, 39)	8.0 (-9.6, 22)	8.3 (2.9, 19)
PINP	19 (-1.1, 34)	-0.29 (-5.9, 7.2)	0.92 (-7.4, 7.1)	4.1 (-12, 21)
U-CTx	20 (4.7, 38)	14 (8.4, 24)	2.9 (-13, 9.0)	8.8 (2.0, 13)
U-NTx	6.8 (1.1, 23)	0.21 (-12, 4.2)	-2.3 (-15, 22)	-0.36 (-13, 19)
Secondary efficacy measure: percentage change from baseline to week 36*				
BAP	5.6 (3.7, 26)	15 (1.7, 29)	1.4 (-6.3, 6.6)	-1.9 (-10, 6.9)
S-CTx	13 (-2.2, 34)	30 (22, 52)	9.2 (-13.5, 22)	13 (1.2, 19)
PINP	15 (-0.14, 39)	24 (-0.33, 56)	10 (0.47, 25)	-0.92 (-16, 22)
U-CTx	17 (-5.8, 43)	46 (26, 70)	7.4 (-28, 29)	23 (1.1, 39)
U-NTx	-6.3 (-20, 5.1)	11 (-1.3, 34)	-1.4 (-14, 46)	6.5 (-6, 26)
Secondary efficacy measure: AUC_0–12 week*_				
BAP, ng/ml × week	1.6 (-2.0, 10)	-3.7 (-8.6, 2.3)	-5.8 (-26, 3.2)	-1.7 (-9.0, 5.7)
S-CTx, ng/ml × week	0.68 (0.52, 1.37)	0.60 (0.28, 0.99)	0.33 (-0.28, 0.73)	0.38 (0.085, 0.65)
PINP, ng/ml × week	42 (25, 73)	-10 (-47, 23)	-11 (-31, 20)	19 (-24, 56)
U-CTx_(adjusted)_, μg/mmol Cr × week	269 (-173, 553)	302 (53, 643)	-242 (-762, 192)	105 (-129, 396)
U-NTx_(adjusted)_, nmol BCE/mmol Cr × week	49 (-2.4, 91)	50 (15, 81)	-6.5 (-49, 30)	0.67 (-92, 42)
Secondary efficacy measure: AUC_0–24 week*_				
BAP, ng/ml × week	17 (-3.7, 29)	-14 (-23, 8.1)	-14 (-55, 9.2)	0.31 (-26, 12)
S-CTx, ng/ml × week	2.0 (1.3, 2.9)	1.5 (1.1, 2.3)	1.0 (-0.41, 1.8)	0.89 (0.046, 1.6)
PINP, ng/ml × week	187 (95, 295)	-6.1 (-57, 79)	19 (-46, 90)	42 (-67, 142)
U-CTx_(adjusted)_, μg/mmol Cr × week	1063 (170, 1868)	1157 (422, 2314)	61 (-1077, 588)	427 (51, 751)
U-NTx_(adjusted)_, nmol BCE/mmol Cr × week	21 (-64, 176)	102 (-49, 210)	-9.0 (-204, 158)	-47 (-265, 58)

*All data are medians (bias-corrected and adjusted 95% bootstrap confidence interval).

AUC, area under the curve; BAP, bone alkaline phosphatase; BCE, bone collagen equivalent; Cr, creatinine; PINP, procollagen type I N-terminal peptide; S-CTx, serum C-terminal telopeptide of type I collagen; U-CTx, urinary C-terminal telopeptide of type I collagen; U-NTx, urinary N-terminal telopeptide of type I collagen.

### Bone-formation markers

**Figure 2:**
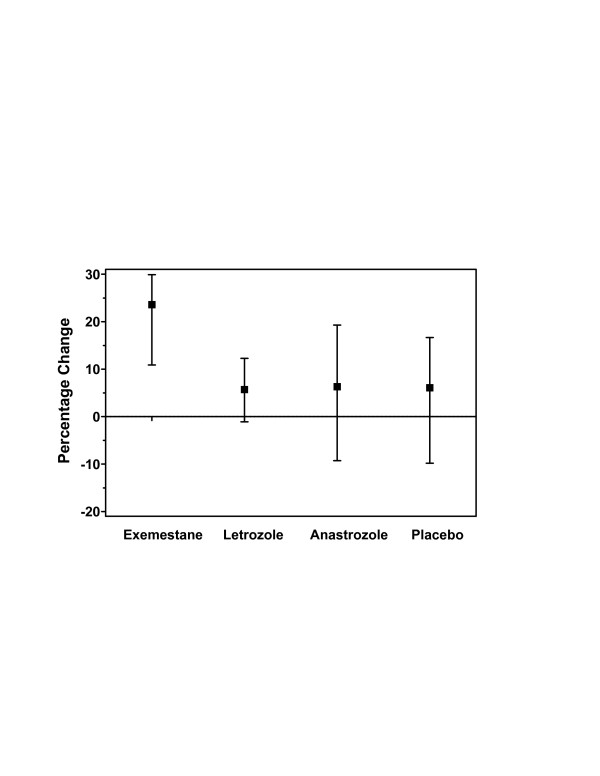
Change in serum concentrations of PINP from baseline to week 24 in the evaluable population. The median percentage change (95% CI) from baseline was consistently positive only for exemestane (24%; 95% CI, 11–30%). The overall differences between the four groups were not statistically significant (*P *= 0.147 using the Kruskall–Wallis test). CI, confidence interval; PINP, procollagen type I N-terminal propeptide.

For the primary end point of the percentage increase from baseline of PINP concentration at 24 weeks, exemestane treatment demonstrated an approximately fourfold greater increase from baseline compared with the other AIs and placebo (Figure 2). Because the magnitude of this change was of interest, a retrospective statistical comparison among treatment arms using a nonparametric Kruskall–Wallis test was performed. The results of this analysis demonstrated that, at 24 weeks, only exemestane treatment consistently resulted in an increase in the level of PINP, with a median percentage change of 24% and 95% CI of 11–30% (Table 3); however, between-group differences did not reach statistical significance at 24 weeks (*P *= 0.147). At week 36, 12 weeks after discontinuation of treatment, the percentage change from baseline was positive for the three AIs, by approximately 24% for letrozole, 15% for exemestane, and 10% for anastrozole, compared with a slight decrease from baseline for placebo.

**Figure 3:**
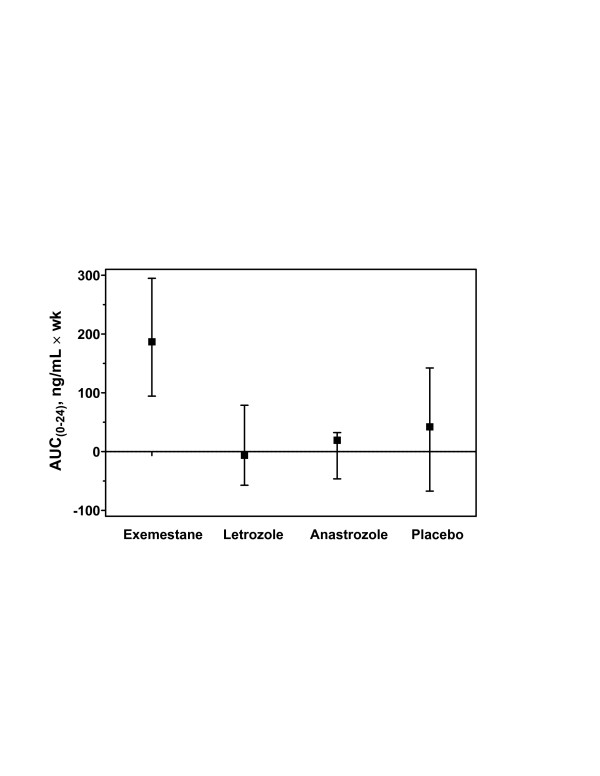
Baseline-adjusted AUC_0–24 week _for serum concentrations of PINP in the evaluable population. The increase in baseline-adjusted AUC_0–24 week _for exemestane was 187% (95% CI, 95–295%). The AUC_0–24 week _for exemestane was significantly greater than anastrozole (*P *= 0.004), letrozole (*P *< 0.001), or placebo (*P *= 0.033). Changes in baseline-adjusted AUC_0–24 week _were not statistically significant for other active treatments or placebo. The overall differences between the four groups were statistically significant (*P *= 0.004 using the Kruskall–Wallis test). AUC, area under the curve; PINP, procollagen type I N-terminal propeptide.

Subjects receiving exemestane exhibited a significantly higher baseline-adjusted AUC for PINP during treatment. Because the differences were significant overall among the four groups (*P *= 0.011 for AUC_0–12 week _and *P *= 0.004 for AUC_0–24 week_), pairwise comparisons were assessed, as above. The AUC_0–12 week _for subjects receiving exemestane was elevated (42%; 95% CI, 25–73%) from baseline, whereas the letrozole- and anastrozole-treated groups showed a decrease of approximately 10% from baseline (*P *= 0.002 and *P *= 0.011 for letrozole or anastrozole vs exemestane, respectively); the placebo group showed an increase of approximately 19%. The AUC_0–24 week _for exemestane was dramatically greater (187%; 95% CI, 95–295%) than that observed for both nonsteroidal AIs (*P *< 0.001 and *P *= 0.004 for letrozole or anastrozole vs exemestane, respectively). Because the magnitude of this change was also of interest, a retrospective statistical comparison, as described above, was again used to compare results between treatment arms (Figure 3).

**Figure 4:**
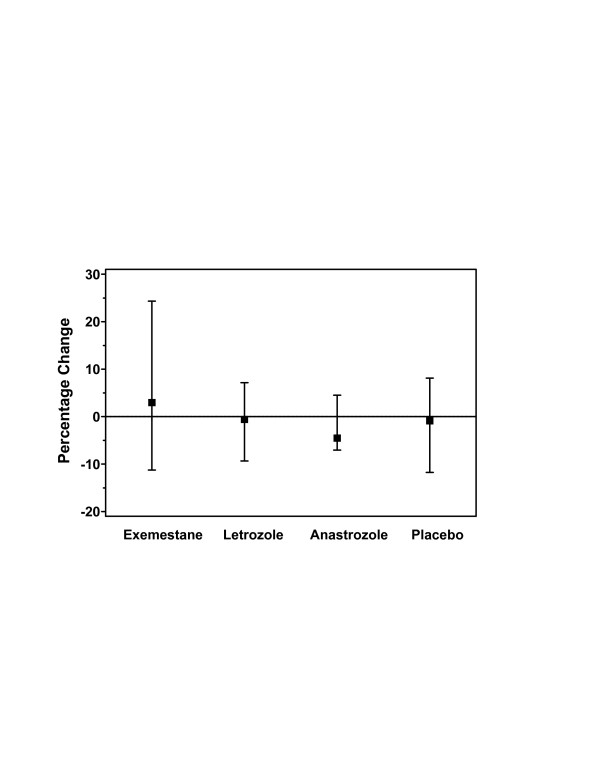
Change in serum concentrations of BAP from baseline to week 24 in the evaluable population. BAP, bone alkaline phosphatase.

The median percentage change in BAP concentration at week 24 in the exemestane-treated group was increased slightly from baseline, whereas other treatment groups exhibited slight decreases in the concentration of BAP from baseline (Figure 4); however, the 95% CI for exemestane was nearly twice that of the other groups at this time point (Table 3). At week 12, all groups exhibited a slight decrease in the concentration from baseline, with anastrozole demonstrating the largest decrease. At week 36, the percentage change in BAP concentration from baseline in the letrozole-treated group was more than twofold compared with the exemestane-treated group and more than tenfold compared with the anastrozole-treated or placebo groups.

For the AUC_0–12 week _and the AUC_0–24 week_, the subjects receiving exemestane were the only group to exhibit an increase from baseline (except the placebo group at 24 weeks); in the other two AI-treated groups, the AUC_0–12 week _decreased approximately 4% from baseline and the AUC_0–24 week _decreased approximately 14% from baseline. Patients administered placebo demonstrated a slight decrease from baseline in the AUC_0–12 week _and their AUC_0–24 week _was essentially unchanged from baseline.

### Bone-resorption markers

For S-CTx, the median percentage change from baseline increased at week 24 with exemestane and letrozole treatment, demonstrating approximately twofold greater increases compared with anastrozole treatment or placebo. These increases were already apparent at week 12, and the increase from week 12 to week 24 was less noticeable. At week 36, the median percentage change from baseline for S-CTx was essentially unchanged from week 24 for the groups receiving letrozole, anastrozole, and placebo, but that of the exemestane-treated group decreased by approximately 35% following the end of treatment at week 24.

The median percentage changes in the AUC_0–12 week _and AUC_0–24 week _for S-CTx were minimal for all groups.

For the median percentage change in U-CTx at week 24, the letrozole-treated group had the largest increase from baseline and 95% CI (34%; 95% CI, 19–74%). Exemestane treatment and placebo increased the level of U-CTx approximately 22% and 15%, respectively, and anastrozole treatment demonstrated only a slight increase (2%; 95% CI, -6% to 29%). By contrast, at week 12, the exemestane-treated group had the greatest increase from baseline (20%; 95% CI, 5–38%) and was approximately sevenfold higher than the anastrozole-treated group, with the letrozole-treated and placebo groups intermediate. At week 36, U-CTx levels remained highly elevated from baseline in the letrozole-treated group (46%; 95% CI, 26–70%) and had an additional relative increase of 37% from the levels observed at the end of treatment. Anastrozole treatment and placebo demonstrated similar patterns of increase, with increases from week 24. By contrast, the level of U-CTx decreased approximately 23% 12 weeks after discontinuation of exemestane treatment.

The AUC_0–12 week _for U-CTx demonstrated large differences between groups, with increases of approximately 275% in the exemestane- and letrozole-treated groups, but a decrease of 242% from baseline in the anastrozole-treated group. The placebo group demonstrated a lesser increase from baseline of 105%. The 95% CIs for all groups were large; the greatest was in the anastrozole-treated group and the least was in the placebo group. For the AUC_0–24 week_, exemestane and letrozole treatment demonstrated extremely large increases from baseline of 1063% and 1157%, respectively, with the group receiving placebo demonstrating a lesser increase of 427% and anastrozole treatment producing an increase of 61% from baseline. The 95% CI for the AUC_(0–24 wk) _in the placebo group was approximately half that of the active-treatment groups.

**Figure 5:**
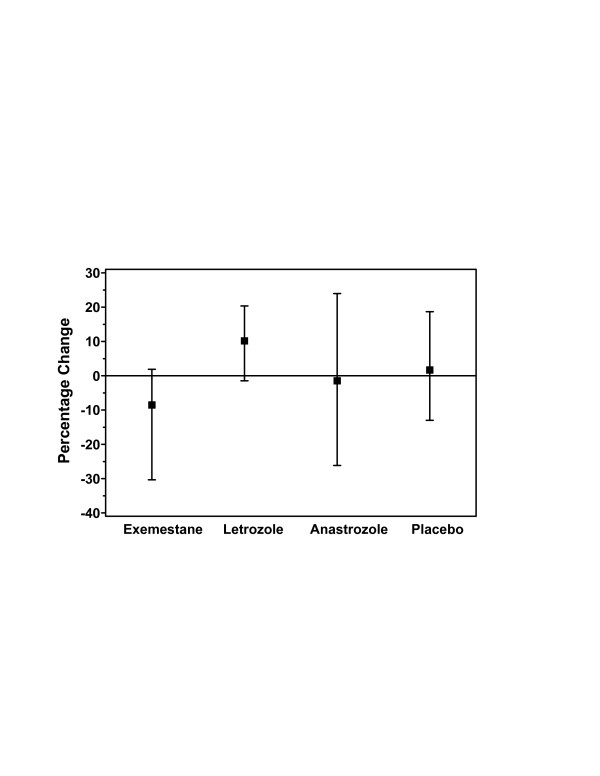
Change in U-NTx concentrations from baseline to week 24 in the evaluable population. UNTx_(adjusted)_, adjusted urinary N-terminal telopeptide of type I collagen.

Changes in the levels of U-NTx were variable across groups and time points. At the primary end point of 24 weeks, letrozole treatment increased the level from baseline, exemestane treatment decreased the level, and anastrozole treatment and placebo exhibited only slight changes in the level from baseline (Figure 5). By contrast, at week 12, the median percentage change in the level after exemestane treatment was increased from baseline, anastrozole treatment demonstrated a slight decrease in the level from baseline, and the level was essentially unchanged in the letrozole-treated and placebo groups. Twelve weeks after discontinuation of study drug, the letrozole-treated and placebo groups again demonstrated moderate increases in the level of U-NTx from baseline, whereas the exemestane- and anastrozole-treated groups exhibited moderate-to-small decreases from the baseline level.

For the AUC_(0–12 week)_, U-NTx was elevated approximately 50% from baseline in the exemestane- and letrozole-treated groups, decreased approximately 6% from baseline in the anastrozole-treated group, and remained essentially unchanged in the placebo group. At 24 weeks, letrozole treatment demonstrated a fivefold greater increase in the AUC_(0–24 week) _from baseline, compared with exemestane treatment, whereas the level in the anastrozole-treated group decreased nearly 10% and that in the placebo group decreased nearly 50% from baseline.

### Estrogen plasma concentrations

Baseline concentrations of estrogens in the mITT population were similar among treatment groups (Table 2). As expected, estrogen concentrations (E_1_, E_2_, and E_1_S) were significantly reduced compared with the placebo group at 12 and 24 weeks in the evaluable subjects treated with all three AIs (*P *< 0.001 for all comparisons; Table 4). The levels of E_1 _and E_2 _decreased to near or below the level of quantification. At week 36 (12 weeks after completion of active treatment), plasma E_1_, E_2_, and E_1_S concentrations had returned to near baseline for the three active-treatment groups and were again similar to those observed in the placebo group.

**Table 4:** Median values and median percentage change from baseline for plasma concentrations of estrogens (mITT population)

**Estrogen**	**Exemestane (*n *= 20)**	**Letrozole (*n *= 20)**	**Anastrozole (*n *= 18)**	**Placebo (*n *= 20)**
**E_1_**				
Baseline, median (min., max.)	31 (11, 72)	28 (13, 51)	29 (14, 61)	26 (9.9, 61)
Week 12, median (min., max.)	2.0 (1.8, 10)	1.8 (1.8, 20)	1.8 (1.8, 84)	31 (8.4, 58)
% baseline, median (95% CI*)	7.8 (6.9, 9.3)	6.6 (5.5, 8.1)	7.6 (6.4, 11)	101 (89, 110)
Week 24, median (min., max.)	1.8 (1.8, 63)	1.8 (1.8, 33)	1.8 (1.8, 44)	26 (7.2, 56)
% baseline, median (95% CI)	6.7 (5.3, 8.6)	6.6 (5.1, 8.1)	7.9 (7.1, 11)	94 (76, 108)
Week 36, median (min., max.)	23 (10, 62)	19 (10, 44)	25 (8.4, 158)	22 (13, 46)
% baseline, median (95% CI)	72 (63, 86)	76 (61, 93)	70 (66, 121)	89 (72, 95)
**E_2_**				
Baseline, median (min., max.)	4.4 (1.1, 13)	4.2 (1.8, 8.6)	3.8 (1.9, 32)	3.9 (1.9, 8.8)
Week 12, median (min., max.)	0.70 (0.70, 2.5)	0.70 (0.70, 2.7)	0.70 (0.70, 104)	3.8 (1.6, 15)
% baseline, median (95% CI)	18 (14, 22)	19 (14, 24)	20 (18, 31)	102 (87, 115)
Week 24, median (min., max.)	0.70 (0.70, 29)	0.70 (0.70, 7.3)	0.72 (0.70, 84)	4.4 (1.9, 12)
% baseline, median (95% CI)	19 (13, 22)	18 (13, 24)	22 (19, 31)	128 (95, 139)
Week 36, median (min., max.)	4.5 (1.6, 36)	4.2 (1.9, 15)	4.2 (1.4, 136)	4.0 (1.6, 11)
% baseline, median (95% CI)	103 (89, 134)	109 (81, 119)	106 (92, 130)	105 (90, 130)
**E_1_S**				
Baseline, median (min., max.)	192 (58, 757)	193 (79, 1075)	202 (64, 896)	200 (62, 1156)
Week 12, median (min., max.)	13 (8.7, 246)	8.7 (6.0, 62)	15 (6.6, 3260)	209 (58, 876)
% baseline, median (95% CI)	9.1 (6.1, 11)	4.5 (3.3, 6.0)	11 (6.7, 13)	110 (91, 146)
Week 24, median (min., max.)	16 (6.1, 839)	10 (6.0, 254)	18 (8.8, 1440)	195 (64, 802)
% baseline, median (95% CI)	11 (7.2, 13)	4.7 (3.0, 7.2)	10 (8.6, 20)	105 (69, 123)
Week 36, median (min., max.)	223 (7.4, 2100)	222 (115, 932)	296 (58, 4890)	205 (68, 864)
% baseline, median (95% CI)	114 (90, 152)	121 (105, 135)	131 (109, 147)	125 (91, 143)

*95% CI, bias-corrected and adjusted 95% bootstrap CI for median.

CI, confidence interval; E_1_, estrone; E_1_S, estrone sulfate; E_2_, estradiol; max; min; mITT, modified intent to treat.

### Lipid profiles

Baseline concentrations of lipids in the mITT population were similar among treatment groups (Table 2). Overall, the percentage changes from baseline to week 12, 24, or 36 in all the lipid parameters monitored were similar between groups (Table 5).

**Table 5:** Median values and median percentage change from baseline for serum lipid profiles (mITT population)

**Lipid**	**Exemestane (*n *= 20)**	**Letrozole (*n *= 20)**	**Anastrozole (*n *= 19)**	**Placebo (*n *= 20)**
**LDL-C (mmol/L)**				
Baseline	4.2	4.2	4.2	3.8
Week 12, % change	-11	-4.5	-12	-6.1
Week 24, % change	-2.6	-4.2	-3.0	2.4
Week 36, % change	1.9	0.25	-5.7	-5.8
**HDL-C (mmol/L)**				
Baseline, median	1.4	1.5	1.4	1.4
Week 12, % change	-4.4	9.0	1.7	-3.5
Week 24, % change	-13	-2.4	-4.4	-6.3
Week 36, % change	-6.9	-2.4	-2.5	-6.8
**Total cholesterol (mmol/L)**				
Baseline, median	6.1	6.1	6.3	5.9
Week 12, % change	-6.8	-1.8	-3.7	-2.6
Week 24, % change	0.78	1.2	-0.68	0.75
Week 36, % change	1.3	-1.9	-5.8	-6.4
**Triglycerides (mmol/L)**				
Baseline, median	1.1	1.2	1.2	1.0
Week 12, % change	-21	6.6	-10	-1.6
Week 24, % change	1.7	4.6	6.3	-7.0
Week 36, % change	-1.1	-5.4	-3.5	-0.28

HDL-C, high-density lipoprotein cholesterol; LDL-C, low-density lipoprotein cholesterol; mITT = modified intent to treat.

### Safety

Treatment-emergent adverse events were reported by 62 (73.8%) subjects, and treatment-related adverse events were reported by 39 (46.4%) subjects. Most adverse events were mild to moderate in severity. Five subjects experienced serious adverse events (two exemestane-, one anastrozole-, and two placebo-treated subjects), none of which were considered related to the study medication. Two subjects (one anastrozole- and one placebo-treated subject) discontinued treatment owing to adverse events, neither of which was considered to be treatment related. The most frequently reported adverse events (>10% of subjects in any treatment group) were headache, alopecia, nasopharyngitis, hot flushes, weight increase, arthralgia, and diarrhea. The incidence of adverse events probably owing to estrogen reduction was comparable between treatment groups.

The incidence of laboratory toxicities was comparable between active-treatment groups and the placebo group. No laboratory abnormality exceeded NCI CTC toxicity grade 2 (moderate). Most of the laboratory abnormalities were grade 1 (mild), and none were considered an adverse event or clinically significant by the investigator.

## Discussion

In postmenopausal women with early breast cancer, AIs offer significant benefit over standard treatment with tamoxifen for 5 years in terms of disease-free survival and, for anastrozole and exemestane, reduced contralateral breast cancer and time to recurrence [20-25]. Despite these encouraging results, the long-term clinical impact of AIs must be established because the aromatase enzyme has an important role in numerous normal physiological processes [26]. A key role of aromatase is local production of estrogens, which are essential for the maintenance of bone integrity [27]. There are, therefore, two important questions that must be addressed, as follows: will AIs accelerate the negative skeletal balance associated with the menopause; and if so, to what extent? Although several relatively short-term clinical studies suggest that AIs accelerate bone loss [2,4-6,8], to date there have been no comparative clinical studies. Because preclinical data suggest that the steroidal AI exemestane might have different effects on bone compared with the nonsteroidal AIs [28], this study was designed to assess the effects of the different AIs by evaluating changes in bone biomarkers after short-term treatment (24 weeks).

This short-term study demonstrates differential effects of the AIs evaluated on bone-turnover markers. For the primary end point of the effect on markers of bone turnover at 24 weeks, markers of bone resorption were increased in all treatment groups, including placebo, but the magnitude of increase was noticeably lower for anastrozole compared with the other groups. Measures of bone resorption (that is to say, S-CTx and U-CTx) were increased to the greatest extent by letrozole; however, the 95% CIs overlapped between all groups. By contrast, exemestane was the only AI to increase the median percentage change from baseline to week 24 for both PINP and BAP concentrations. Although the PINP concentration was increased from baseline by approximately 6% in all other groups, the BAP concentration decreased from baseline or remained essentially unchanged. A retrospective statistical comparison of the changes in PINP levels showed a statistically greater increase for exemestane treatment compared with the other AIs or placebo at week 24, but this statistical comparison must be interpreted in light of its retrospective nature and the potential lack of sufficient power of this small study.

Following discontinuation of treatment at 24 weeks, the levels of markers of bone resorption (S-CTx and U-CTx) remained elevated or continued to increase from baseline to week 36 in patients receiving letrozole, anastrozole, or placebo; by contrast, the levels of markers of bone resorption tended to decline towards baseline at week 36 following discontinuation of exemestane at week 24. The effect of exemestane withdrawal for 3 and 6 months has been studied in postmenopausal women following 2 years' treatment [29]. After exemestane withdrawal, bone-resorption markers returned to, or below, baseline values within 6 months, with the exception of the S-CTx concentration. For markers of bone formation (for example, BAP, PINP, and osteocalcin), a successive decrease in values at 3 and 6 months after terminating therapy was recorded; however, the levels of all markers of bone formation remained elevated at 6 months compared with the baseline value. The results of the current study, described after 12 weeks of exemestane withdrawal, are consistent with these observations. These findings could be further assessed by correlating BMD measurements and fracture data to changes in bone biomarkers in a large confirmatory trial.

The primary benefit of the use of biochemical markers is their ability to reflect small changes in bone formation or resorption before such changes are detectable radiologically [30]. Although bone-turnover data are typically reported at discrete time points, we suggest that assessing data over time using the AUC method might be more illustrative of the true impact of interventions. This technique avoids the oversimplification associated with reporting a value at a single point in time, which might lead to incorrect inferences. Instead, the AUC provides a view of the whole spectrum of values because results are depicted over time [19,30,31].

In our study, the data gathered by the AUC analysis demonstrate that exemestane might have a greater effect on bone formation compared with the nonsteroidal AIs, particularly PINP levels. By contrast, letrozole, the more potent of the two nonsteroidal agents [32,33], demonstrated the greatest impact on bone resorption, which continued to be significantly elevated 3 months after discontinuation of treatment. Thus, this short-term study suggests there are differences between steroidal and nonsteroidal AIs; however, the results from this exploratory, short-term study must be replicated by a larger controlled trial of longer duration.

The basis for the hypothesized difference in the effects of nonsteroidal AIs and the steroidal aromatase inactivator exemestane is the steroidal structure of exemestane and the androgenic activity of its primary metabolite, 17-hydroexemestane [18]. Both androgens and estrogens have a role in skeletal development and maintenance in women [27]. However, estrogens reduce bone resorption, whereas androgens stimulate bone formation and are thought to be effective in reducing fracture risk [34]. In fact, regardless of estrogen status, perimenopausal women with higher androgen concentrations have a slower rate of bone loss compared with women who have lower concentrations [35], and administration of low doses of oral androgen in combination with estrogen results in higher concentrations of bone-formation markers than treatment with estrogen alone in postmenopausal women [36]. On the basis of its pharmacologic characteristics, these findings support the hypothesis that exemestane might have differential effects on bone compared with nonsteroidal AIs.

When the bone-marker data were analyzed for the mITT population (data not shown), the results were similar to those observed in the evaluable population. The mITT population included all subjects who received at least one dose of study medication and had baseline and week 12 assessments at least. Therefore, unlike the evaluable population, the mITT population included the five patients who did not demonstrate estrogen suppression following treatment with an AI. Because third-generation AIs provide consistent suppression of estrogen synthesis in postmenopausal women, it is our impression that these five subjects had poor or inconsistent compliance and were, therefore, not evaluable for the purpose of our study because our analysis was designed to demonstrate the potentially unique response of bone biomarkers to AIs in the presence of effective suppression of estrogens.

We did not report the BMD or fracture rate because of the relatively short duration of our study. BMD is directly related to fracture risk and, therefore, often used to diagnose osteoporosis. However, the BMD must be monitored for several years, not months, to be useful for determination of fracture risk, and changes in BMD do not correlate linearly to fracture risk reduction. Moreover, BMD is only one factor contributing to bone strength and fracture risk. Bone strength is determined by bone quantity (that is to say, density and size) and bone quality (that is to say, microarchitecture and macroarchitecture, material properties, and turnover) [37]. In mechanical terms, the load-bearing capacity or quality of bone is determined by ultimate force (strength), resilience, stiffness, and toughness, and the overall quality of bone is affected by the rate of bone turnover [38]. Although BMD is considered a good diagnostic tool for identifying osteoporosis in untreated patients, emerging data suggest that biochemical markers of bone turnover might also be accurate predictors of treatment-induced bone changes [37,38].

Ultimately, adverse effects on bone metabolism are among the most serious concerns when considering the long-term use of AIs in postmenopausal women. Although measures such as exercise, calcium and vitamin D supplementation, and bisphosphonate administration are being used to alleviate this risk, a finding that exemestane might be superior to other third-generation AIs, in terms of its effects on bone metabolism, could have clinical significance. For this reason, two large ongoing trials are of particular interest: the CAN-NCIC-MA27 study and its companion study, evaluating BMD in postmenopausal women who have primary breast cancer that is treated using exemestane or anastrozole, and the Femara Anastrozole Clinical Evaluation study, a comparison of letrozole with anastrozole in the treatment of postmenopausal women with hormone-receptor-positive and node-positive breast cancer. These studies should help determine the relative differences in the risk of bone loss with long-term use of the different AIs.

Our data support the hypothesis that a mild androgenic effect on bone can be exerted by the steroidal AI exemestane. Our study shows a qualitative difference in the concentration of the bone-formation marker PINP in a short-term, 24-week treatment period, but it involved a small number of women and these findings need confirmation by a larger trial. It is also important to note that all AIs are associated with bone loss, and only long-term comparative clinical studies monitoring fracture outcome will determine the relative effects of the different AIs on fracture risk. Changes in the levels of bone-resorption markers are more sensitive in predicting subsequent fracture risk than changes in the levels of bone-formation markers, thus limiting the interpretation of our short-term findings [39].

However, because androgens affect bone quality and quantity [40,41], the use of surrogate markers, such as BMD, to assess bone effects of AIs might not provide a clear indication of comparative fracture risk. Bone-quality studies using bone biopsies and histomorphometry might prove valuable for assessing the early effects of exemestane on bone quality.

## Conclusion

In recent years, use of the third-generation AIs anastrozole, letrozole, and exemestane has resulted in improved clinical outcomes for postmenopausal patients with early breast cancer. However, because of their estrogen-lowering effect, AIs might increase the risk of osteoporosis and fractures in some patients. Preclinical studies have suggested that exemestane (a steroidal aromatase inactivator) might have differential effects on bone compared with the nonsteroidal AIs anastrozole and letrozole. In our study, exemestane was the only AI that significantly increased serum levels of PINP, a marker of bone formation, at 24 weeks in healthy postmenopausal women. These data need confirmation by a larger comparative trial. Whether these observations correlate to a decreased relative risk of osteoporosis and fractures compared with nonsteroidal AIs in patients receiving long-term adjuvant treatment for early breast cancer requires further study.

## List of Abbreviations

AI = aromatase inhibitor; AUC = area under the curve; BAP = bone alkaline phosphatase; BMD = bone mineral density; CI = confidence interval; CTx = C-terminal telopeptide of collagen type I; E_1 _= estrone; E_1_S = estrone sulfate; E_2 _= estradiol; ELISA = enzyme-linked immunosorbent assay; HPLC = high-performance liquid chromatography; mITT = modified intent to treat; NCI CTC = National Cancer Institute Common Toxicity Criteria; NTx = N-terminal telopeptide of type I collagen; PINP = procollagen type I N-terminal propeptide; QUS = quantitative ultrasonometry; S-CTx = serum C-terminal telopeptide of type I collagen; U-CTx = urine-adjusted urinary C-terminal telopeptide of type I collagen; U-NTx = urine-adjusted urinary N-terminal telopeptide of type I collagen.

## Competing interests

PEG has served as a speaker for, and received advisory honoraria from, Novartis, Pfizer, and AstraZeneca. PH has received unrestricted educational grants, consultancy fees, and research funding from AstraZeneca, Eli Lilly, GlaxoSmithKline, Novartis Oncology, Pfizer Oncology, Procter & Gamble, and Roche. PA and MS are employees of Pfizer Inc. TT and JB-B have no competing interests. The article processing charge assessed by the journal was paid by Pfizer Inc.

## Authors' contributions

PEG conceived of the study, participated in its design, and helped to draft the manuscript. PH participated in the design of the study. PA conducted the statistical analysis. MS and PA participated in data analysis, data interpretation, and drafting of the manuscript. TT and JB-B were the principal investigators responsible for all clinical aspects of this study. All authors read and approved the final manuscript.
